# Integer-Linear-Programing Optimization in Scalable Video Multicast with Adaptive Modulation and Coding in Wireless Networks

**DOI:** 10.1155/2014/769241

**Published:** 2014-09-03

**Authors:** Dongyul Lee, Chaewoo Lee

**Affiliations:** School of Electrical and Computer Engineering, Woncheon Hall 432, Ajou University 206, World Cup-ro, Yeongtong-gu, Suwon-si, Gyeonggi-do 443-749, Republic of Korea

## Abstract

The advancement in wideband wireless network supports real time services such as IPTV and live video streaming. However, because of the sharing nature of the wireless medium, efficient resource allocation has been studied to achieve a high level of acceptability and proliferation of wireless multimedia. Scalable video coding (SVC) with adaptive modulation and coding (AMC) provides an excellent solution for wireless video streaming. By assigning different modulation and coding schemes (MCSs) to video layers, SVC can provide good video quality to users in good channel conditions and also basic video quality to users in bad channel conditions. For optimal resource allocation, a key issue in applying SVC in the wireless multicast service is how to assign MCSs and the time resources to each SVC layer in the heterogeneous channel condition. We formulate this problem with integer linear programming (ILP) and provide numerical results to show the performance under 802.16 m environment. The result shows that our methodology enhances the overall system throughput compared to an existing algorithm.

## 1. Introduction

Rapid advancement in mobile wideband wireless network (i.e., 4G and 5G) supports real time services such as IPTV and live video streaming. Nevertheless, efficient resource allocation is required to satisfy delay requirements and to overcome the bandwidth shortage due to the increased number of users. Because of the sharing nature of the wireless medium, the wireless multicast has attracted much attention when multiple users want to receive the same video service at the same time [1].

Multicast service in wireless heterogeneous networks can adaptively choose a certain modulation and coding scheme (MCS) [2] based on the channel status and the hardware capability of the receivers. For the nonscalable coding (or called a single-layer coding) the multicast data rate and video quality are determined by the user who experiences the worst channel status [1, 3].

To alleviate the video performance degradation caused by the user who has the worst channel status, scalable video coding (SVC) [4] with adaptive modulation and coding (AMC) provides an excellent solution. In SVC, a video stream is divided into multiple layers. SVC encodes video with the nested dependency: the base layer encodes the basic video quality and higher layers, called the enhancement layers, refine the visual quality from the base layer with smaller quantization granularities. To enhance the overall performance of wireless multicast video streaming, we assign a low MCS to base layer and high MCSs to enhancement layers, so that the users in bad channel conditions receive fewer enhancement layers and obtain basic video quality, while the users in good channel conditions receive more enhancement layers and obtain better video quality.

A key issue arising under this setting is how each layer selects its MCS under the constraint of available radio resources for multicast services. A lot of research has studied the resource allocation problem in wireless multicast streaming video services [5–10]. In particular, [8, 10] are most closely related to our research work, which provides solutions in both the single channel and slot TDMA-based networks. Reference [8] proposed an algorithm that guarantees full coverage which provides all destinations with at least a base layer to accommodate them. It allocates other MCSs to the enhancement layers with a heuristic algorithm to maximize the sum of utilities of all the receivers. It assumed that the size of each video layer is the same and the utility function of a user is simply a log function of the received data. The size of each layer is usually different, such that SVC supports various video qualities. The video quality experienced by a user is peak signal-to-noise ratio (PSNR) not a simple log function, so PSNR should be used in the resource allocation.

Reference [10] formulated a model that provides different size for each layer to support various video qualities. It used PSNR as a utility function to reflect the video quality experienced by a user and utilized dynamic programming for optimal network design. While the PSNR depends on the number of received packets in a layer, it increases only when all the packets in a layer are received fully. This PSNR model cannot be applied to other encoding methods of which PSNR increases with respect to the received packets. Hence, the PSNR which is the function of the received packets has to be derived. To search for an optimal solution easily, it assumes that the packet size and the slot size are fixed. However, if data rate is varied, the fixed-size slot cannot be utilized fully. Thus, if the slot size is determined based on each MCS, more video data can be transmitted without idle time in a slot.

To overcome these problems, we analyze PSNR models of various SVC encoding methods and choose a utility function to accommodate all the PSNR models. We also formulate a model which varies the slot size based on each MCS and utilize integer linear programming (ILP) to obtain the optimal solution.

The rest of the paper is organized as follows. The background and system architecture are discussed in Section 2. In Section 3, we present the system model and utility formulation. The problem formulation is described in Section 4.  Section 5 explains ILP modeling and Section 6 shows the numerical results. Finally, Section 7 concludes the paper.

## 2. Background

### 2.1. Scalable Video Coding

Scalable video coding compresses a raw video sequence into a base layer and one or multiple enhancement layers. The base layer provides video data whose resolution is low, while the higher layers refine the video generated by the lower layer. SVC can provide adaptive video quality in wireless networks, but its performance is poor in terms of video quality compared to single-layer coding. Recently, SVC has improved its coding efficiency. For example, the newly established MPEG-4 SVC provides equal visual quality comparable with H.264/AVC (single-layer coding) but with at most 10% higher bit rate.

**Figure 1:**
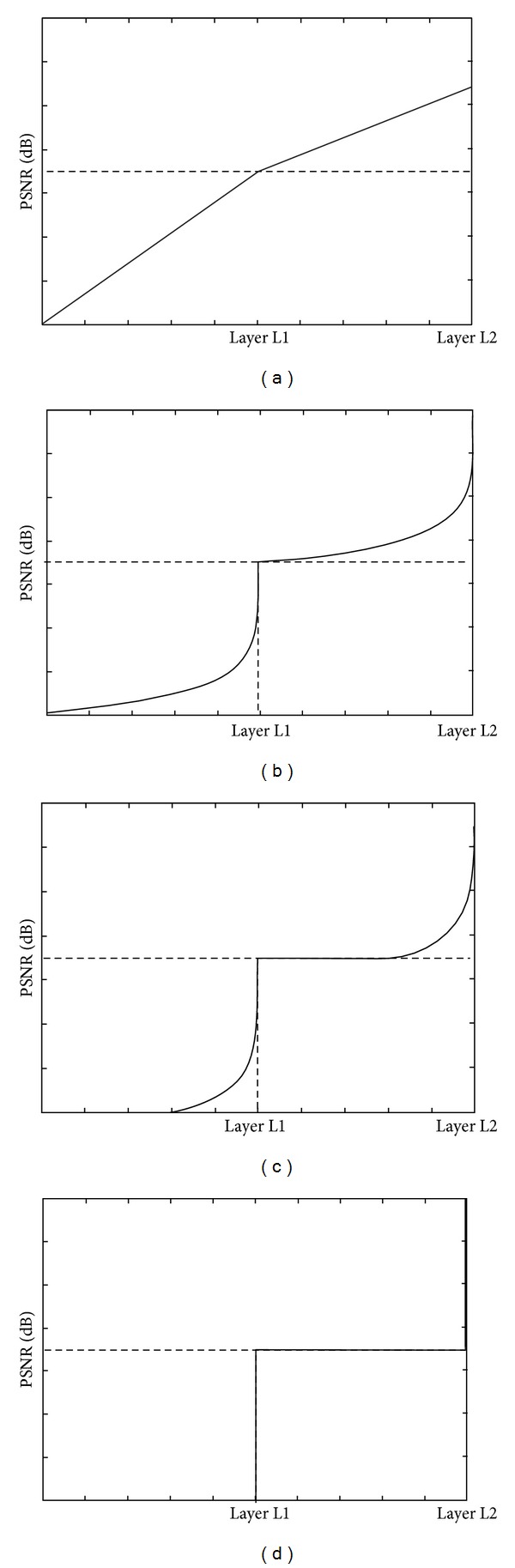
PSNR to the number of the packets received at a user in the four schemes.

The quality of video can be measured using PSNR, which is a nondecreasing function of the received video data [11–15]. The PSNR function has different shapes according to encoding schemes, and it can be classified into four groups as illustrated in Figure 1 [11–15]. From Figure 1, we can observe that the functions of Figures 1(a) and 1(d) are represented as piecewise linear functions directly. However, Figures 1(b) and 1(c) show nonlinear functions, and so we approximate them to piecewise linear functions in the next section.

### 2.2. Mobile Wireless Systems

**Figure 2:**
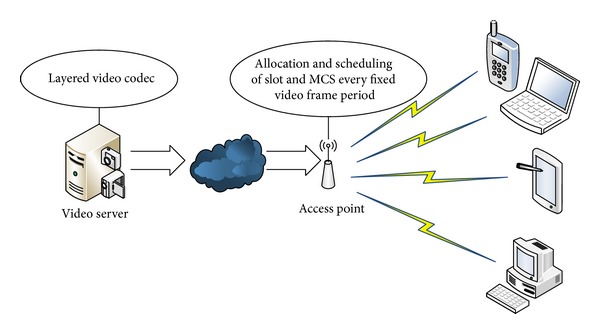
System architecture for scalable video multicast.

In infrastructure-based wireless networks, we consider the video multicast. A video server is equipped in the layered video codec to encode the video into multiple layers as illustrated in Figure 2. The video server then streams the video from all layers to APs (or base stations). The number and the size of the layers corresponding to each video frame are delivered to APs for every video frame period. The connection speed between the video server and the APs (or base stations) is high and reliable. Video stream is usually transmitted at a constant bit rate and an AP or base station forwards the packets delivered by video server to users for fragmentation. So, the packet size is fixed and is transmitted in a wireless channel.

Scheduling (resource allocation) in a wireless network MAC is done once for every scheduling frame period whose length is identical to the length of the video frame in our system. A scheduling frame consists of multiple time slots. The size of a time slot is the minimum time required to transmit a packet with a MCS. It means that the maximum number of available slot sizes is equal to the number of MCSs.

We consider the heterogeneous wireless networks, and so the users who experience relatively bad channel status can select a relatively high rate that leads to the considerable packet loss. In our system, each user measures its SNR and finds which MCS indicates the maximum data rate that satisfies target BER (bit error ratio) to decode the received data. An AP or base station assigns the radio resource and MCSs to layers to maximize the sum of utilities of all users to find the optimal solution.

## 3. System Model and Utility Formulation

We consider the one-hop broadcasting in wireless networks for the problem formulation. There are *J* users, and *F* is the size of a scheduling frame period, which is equal to that of video frame period. We assume a set of possible MCSs *M* = {1,…, *M*} (e.g., MCS *m* = 1 indicates QPSK-1/2 and others). High MCS *m* means a high data rate. If a user can decode the data provided by MCS *m* by satisfying a target BER, it can also decode the data that is less than MCS *m*. *M*_*j*_ ∈ *M* is the maximum MCS, which can be received by user *j* based on the channel status. User *j* transfers its MCS *M*_*j*_ ∈ *m* to its AP (base station). *t*_*m*_ is the time slot size given by MCS *m*. We consider a single multicast session which has *L* layers. Each layer is divided into many slices, which is also split into the packets of fixed-size. *N*_*l*_ is the number of packets of layer *l*.

Now, we explain a utility function and the related variables. As aforementioned, the satisfaction of the users is proportional to the quality of multimedia defined as PSNR, which is a function of the received throughput. We assume that there is no packet loss in the wireless channel. That is, user *j* can decode a packet if it is transmitted by MCS *m*′ ≤ *M*_*j*_. As illustrated in Figure 1, the PSNR function has four types. First, we are focusing on the second scheme of Figure 1(c) because the other schemes can be easily explained using this.

**Figure 3:**
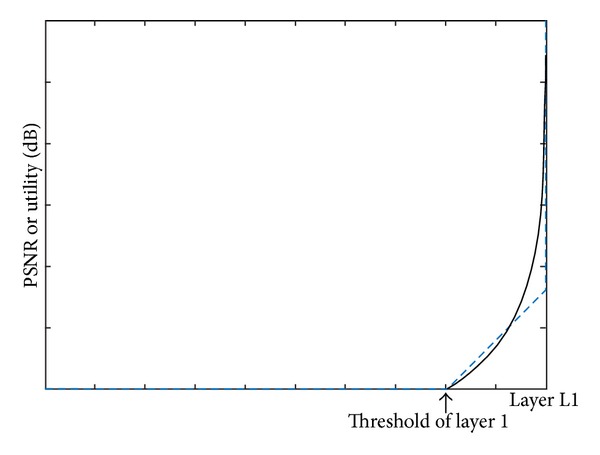
The difference between the original PSNR and approximated PSNR in a couple of packet losses.

As we can see in Figure 1(c), it is nonlinear function and ILP cannot be formulated. In this paper, we approximate it with piecewise linear function as illustrated in Figure 3. We can see that there is relatively large difference between the original PSNR and the approximated one with a few packet losses. However, in case of more packet loss, it shows little difference. In fact, the packet loss which shows relatively large difference is quite less among all the packets and so it is not critical. Although this is incomplete, it can guarantee higher total utility than those of the existing methods [8–10] because the existing methods just consider a utility model that increases only when a user receives a layer completely. This approximation also can be formulated by ILP modeling. Figure 3 shows that the function has zero utility until it reaches the threshold, indicating the number of packets *δ*_*l*_ which does not increase the video quality in a layer. Let *α*_*l*,*m*_ be the number of received packets which have zero utilities in layer *l*. The utility function increases linearly when the packets in a layer *l* are received from the threshold *δ*_*l*_ to the number of packets *N*_*l*_ of layer *l*′. Let *β*_*l*,*m*_ be the number of received packets which increases the utility linearly. Denote *y*_*l*,*m*_ as the indicator function that is 1 if layer *l* is transmitted by ∀*m*′ ≤ *m* and 0 otherwise:

(1) $$\begin{array}{c} y_{l,m}=\{\begin{array}{cc} 1, & \text{if}\;\overset{m}{\underset{m^{\prime }=1}{\sum }}\alpha_{l{,m}^{\prime }}=\delta_{l},\\ 0, & \text{otherwise}. \end{array} \end{array}$$

Denote *z*_*l*,*m*_ as the indicator function that is 1 if all the received packets *β*_*l*,*m*_ are modulated by ∀*m*′ ≤ *m* and 0 otherwise:

(2) $$\begin{array}{c} z_{l,m}=\{\begin{array}{cc} 1, & \text{if}\;\overset{m}{\underset{m^{\prime }=1}{\sum }}\beta_{l,m^{\prime }}=N_{l}-\delta_{l},\\ 0, & \text{otherwise}. \end{array} \end{array}$$

Here, we set that *z*_*l*,0_ = 0 because *β*_*l*,0_ = 0. Hence, with these indicator variables, we utilize the approximated PSNR model as the utility function of the user *j* given as

(3) $$\begin{array}{c} U_{j}=\overset{L}{\underset{l=1\;}{\sum }}\overset{M_{j}}{\underset{m=1}{\sum }}\left(r_{m}t_{m}\phi_{l}\beta_{l,m}+\varphi_{l}\left(z_{l,m}-z_{l,m-1}\right)\right) \end{array}$$

with the constraints of layer transmission:

(4) $$\begin{array}{c} \overset{M_{j}}{\underset{m=1}{\sum }}\alpha_{l,m}\leq \delta_{l},\;\forall l,\\ \overset{M_{j}}{\underset{m=1}{\sum }}\beta_{l,m}\leq N_{l}-\delta_{l},\;\forall l,\\ 0\leq \overset{M_{j}}{\underset{m=1}{\sum }}\alpha_{l,m}+\overset{M_{j}}{\underset{m=1}{\sum }}\beta_{l,m}\leq N_{l},\;\forall l, \end{array}$$

where *ϕ*_*l*_ is the PSNR weight of the packets which increases the utility of layer *l* linearly. *φ*_*l*_ is the PSNR weight of the complete reception of layer *l*. *r*_*m*_ *t*_*m*_ *ϕ*_*l*_ *β*_*l*,*m*_ in (3) means the approximated PSNR to *β*_*l*,*m*_ in layer *l*. *φ*_*l*_(*z*_*l*,*m*_ − *z*_*l*,*m*−1_) in (3) means the approximated PSNR by the complete reception of layer *l*. That is, if the last packet of layer *l* is transmitted by MCS *m*′, (*z*_*l*,*m*_ − *z*_*l*,*m*−1_) has 1 only if *m* = *m*′, otherwise zero.

Three other methods of the utility function can be easily derived as follows. If we set *δ*_*l*_ = 0, the first scheme can be formulated. If we set *δ*_*l*_ = 0 and *φ*_*l*_ = 0, the third scheme can be formulated. If we set *φ*_*l*_ = *N*_*l*_, the final scheme can be formulated.

## 4. Problem Formulation

In this paper, we formulate an ILP problem to assign modulation and coding scheme for each layer in terms of the sum of utilities under any given resource constraints. That is, we have to find out

(5) $$\begin{array}{c} \alpha =\left[\begin{array}{ccc} \alpha_{1,1} & \cdots & \alpha_{1,M}\\ \vdots & \ddots & \vdots \\ \alpha_{l,1} & \cdots & \alpha_{l,M} \end{array}\right],\;\;\beta =\left[\begin{array}{ccc} \beta_{1,1} & \cdots & \beta_{1,M}\\ \vdots & \ddots & \vdots \\ \beta_{l,1} & \cdots & \beta_{l,M} \end{array}\right] \end{array}$$

in order to

(6) $$\begin{array}{c} \text{Maximize}\;\underset{\forall j}{\sum }U_{j} \end{array}$$

following the total time constraints:

(7) $$\begin{array}{c} 0\leq \overset{L}{\underset{l=1\;}{\sum }}\overset{M}{\underset{m=1}{\sum }}t_{m}\alpha_{l,m}+\overset{L}{\underset{l=1\;}{\sum }}\overset{M}{\underset{m=1}{\sum }}t_{m}\beta_{l,m}\leq F \end{array}$$

and layer transmission constraints (1), (2), and (4). Since this formulation does not consider the nested dependency of SVC, it cannot solve the optimal solution yet. We present three lemmas to get an optimal solution.

**Lemma 1 .**

In the optimal solution of the multicast resource allocation problem in wireless networks with the encoding methods [11], if the last packet of layer *l* − 1 is transmitted using modulation *m*′, the packets that have zero utility in layer *l* use *m*, and then it must be *m* ≥ *m*′. That is, the following condition should be satisfied:

(8) $$\begin{array}{c} 0\leq \alpha_{l,m}\leq \delta_{l}z_{l-1,m} \end{array}$$

with constraint of *z*_*l*,*m*_, (2).

**Proof**

We assume that two layers *l* and *l*′ use modulations *m* and *m*′, respectively. If *l* > *l*′ and *m* < *m*′, one can improve the utility by swapping *m* and *m*′ because the utility of packets of layer *l* using *m* is less than and equal to the utility of layer *l* using *m*′.

**Lemma 2 .**

In the optimal solution of the multicast resource allocation problem in wireless networks with the encoding methods [11], if the last packet which has zero utility in layer *l* is transmitted using modulation *m*′, the packets that have nonzero utilities in layer *l* use *m*; then, it must be *m* ≥ *m*′. That is, the following condition should be satisfied:

(9) $$\begin{array}{c} 0\leq \beta_{l,m}\leq \left(N_{l}-\delta_{l}\right)y_{l,m} \end{array}$$

with constraint of *y*_*l*,*m*_, (1).

**Proof**

We assume that packets which have zero utilities and packets which increase the utility linearly of layer *l* use *m* and *m*′, respectively: *α*_*l*,*m*_ and *β*_*l*,*m*′_. If *m* < *m*′, one can improve the utility by swapping *m* and *m*′ because the utility of *β*_*l*,*m*_ is less than and equal to the utility of *β*_*l*,*m*′_.

Lemmas 1 and 2 mean that a MCS can be assigned sequentially in the ascending order. We define full coverage property which guarantees that all the users receive the minimum video quality in Lemma 3.

**Lemma 3 .**

Let *J*_*m*_ be the set of users that can receive MCS *m*. To accommodate all users, the base layer should be transmitted by the MCS *m*_*base*_ = max⁡⁡{*m* : *J*_*m*_ = *J*}:

(10) $$\begin{array}{c} \alpha_{1M_{base}}+\beta_{1M_{base}}=N_{1}. \end{array}$$

**Proof**

The proof follows from the fact (1) that all users have to access the base layer and (2) the time resource required to fit the base layer will diminish with increasing modulation.

Nevertheless, since (1) and (2) have nonlinear properties, they should be changed as the linear equations to formulate ILP problem.

## 5. ILP Modeling

We develop the remaining works to change conditions (1) and (2) as ILP model. The following lemma is proposed to change as the linear equations.

**Lemma 4 .**

For *i* ∈ {0,1,…, *I*}, *c*_*i*_ ∈ {0, *N* ^+^}, if binary random variable *x*_*i*_ ∈ {0,1} has the following condition:

(11) $$\begin{array}{c} x_{i}=\{\begin{array}{cc} 1, & if\;\;\overset{I}{\underset{i=1}{\sum }}A_{i}B_{i}c_{i}=D,\\ 0, & otherwise, \end{array} \end{array}$$

where *A*_*i*_ and *B*_*i*_ are the elements of a constant vector and *D* is a constant and is always larger than ∑_*i*=1_^*I*^ *A*_*i*_ *B*_*i*_ *c*_*i*_, conditional equation (11) is given as

(12) $$\begin{array}{c} Dx_{i}\leq \overset{I}{\underset{i=1}{\sum }}A_{i}B_{i}c_{i}, \end{array}$$

(13) $$\begin{array}{c} \overset{I}{\underset{i=1}{\sum }}A_{i}B_{i}c_{i}-D<x_{i}. \end{array}$$

**Proof**

We can prove it easily. Equation (12) means that *x*_*i*_ should be 0 if ∑_*i*=1_^*I*^ *A*_*i*_ *B*_*i*_ *c*_*i*_ ≠ *D*, and it can be 1 or 0 otherwise. On the contrary to this, (13) means that *x*_*i*_ should be 1 if ∑_*i*=1_^*I*^ *A*_*i*_ *B*_*i*_ *c*_*i*_ = *D*, and it can be 1 or 0 otherwise. If we use these two equations, (12) and (13), we can guarantee condition (11). Using Lemma 4, the conditions in (1) and (2) are changed as follows.

**Theorem 5 .**

*y*_*l*,*m*_ should be satisfied by

(14) $$\begin{array}{c} \delta_{l}y_{l,m}\leq \overset{m}{\underset{m^{\prime }=1}{\sum }}\alpha_{l,m^{\prime }},\\ \overset{m}{\underset{m\prime =1}{\sum }}\alpha_{l,m^{\prime }}-\delta_{l}<y_{l,m}. \end{array}$$

**Proof**

This can be easily proved by Lemma 4.

**Theorem 6 .**

*z*_*l*,*m*_ should be satisfied by

(15) $$\begin{array}{c} \left(N_{l}-\delta_{l}\right)z_{l,m}\leq \overset{m}{\underset{m^{\prime }=1}{\sum }}\beta_{l{,m}^{\prime }}\\ \overset{m}{\underset{m^{\prime }=1}{\sum }}\beta_{l{,m}^{\prime }}-\left(N_{l}-\delta_{l}\right)<z_{l,m}. \end{array}$$

**Proof**

This is can be easily proved by Lemma 4.

The ILP problem to maximize the sum of utilities of all users in the wireless multicast service streaming can be formulated as

(16) $$\begin{array}{c} \arg\underset{\alpha ,\beta }{\max}\underset{\forall j}{\sum }U_{j} \end{array}$$

with the constraints (4), (7), (8), (9), (10), (14), and (15).

In the worst case, since our method utilizes the same domain as the existing method [10] to find out an optimal solution, the complexity obtained *O*(*JL* + *LM* ^2^ *F*/*t*_*M*_). *O*(*JL*) is the complexity which is precalculated to find out the highest MCS to each node. *O*(*LM* ^2^ *F*/*t*_*M*_) is the complexity to solve **α** and **β** for all 1 ≤ *l* ≤ *L*, 1 ≤ *m* ≤ *M*, and 1 ≤ *m* ≤ *M*. *F*/*t*_*M*_ is the maximum number of slots and *t*_*M*_ is the minimum size of a slot. The complexity of the proposed method is pseudopolynomial due to the factor related to the time resource and the number of nodes. Since the complexity is linearly proportional to the number of nodes, the network size does not deteriorate the performance in the proposed methods. Therefore, the complexity of the proposed method is acceptable.

## 6. Numerical Results

In this section, we present the numerical results for our proposed solution. We consider one base station with 14 mobile users distributed in the circular area from base station. The test is based on the 802.16 m evaluation methodology document [16] for a 3.5 MHz spectrum in 3.5 GHz range. We assume that there are four types of MCSs in the IEEE 802.16 m standard [17].  Table 1 shows the MCSs we use in our test and the number of users who can support each MCS. We assume that, for every scheduling period, the fixed-size packet of 60 bits is transmitted in a wireless channel. Other various settings including MAC/PHY header, ACK, interframe space time, and others are not considered. This is because we aim to provide abstraction of important features of our solutions. Other existing researches [8, 10] are also not considered for it. If the setting is required, our solution can use it easily.

In order to analyze the features of our solution, we consider a single video session. Each video session has a fixed layer rate of 800 kb/s, and the number of layers in each session is 8. This is based on the standard of the SVC extension [4] of H.264/MPEG4-AVC. So, each layer can handle 400 packets in a scheduling period because the size of the layer is 30 Kbyte in a scheduling period. We set that *ϕ*_*l*_ = {1,0.95,0.9,…, 0.65} and *φ*_*l*_ = {10,10,…, 10} for *l* = {1,…, 8} based on [13].

We compare our solution with the fixed-slot solution which was solved by dynamic algorithm [10], as the scheduling varies period from 20 ms to 30 ms with interval 1.25 ms. The software package CPLEX is used to find the solution.  Table 2 shows the size of a slot to transmit a packet in both solutions and the number of packets that can be transmitted by the method during a slot of the fixed-slot method.

**Figure 4:**
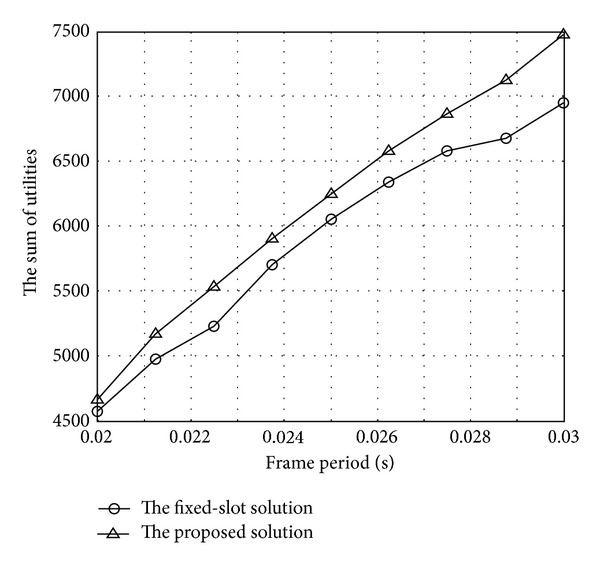
The sum of utilities with varying frame period.

Figure 4 shows the sum of utilities of two solutions. The proposed method enhances the sum of utilities by about 1.9–7.6% compared to the other method. The difference between the two solutions shows an increasing trend. This is because the proposed method assigns more time resource to the low MCSs compared to the other method by reducing the idle time. By detailed observation at 30 ms of frame period, we can understand why the proposed method can enhance the performance.  Table 3 specifies the performance difference between the proposed solution and the fixed-size solution at 30 ms of frame period. The sum of utilities of our solution is 6950, while that of the fixed-slot solutions is 7478. Our solution is higher about 7.6%. This is because by eliminating idle time our solution provides second MCS (*m* = 2) with more slots than those provided by the fixed-slot solution even though both provide the transmission of all the layers.

## 7. Conclusion

In this paper, we considered and studied the resource allocation problem in SVC video multicast with AMC in wireless networks. First, we assume that all packets have equal length to consider the real video transmission environments. It is also assumed that each MCS has different slot length, which is the minimum time length required to transmit a packet with the corresponding MCS. We formulated a utility function as a piecewise function for ILP modeling using the existing results [11, 13]. Finally, we define ILP problem by proposing some lemmas and a theorem to formulate ILP problem. We provide numerical results to show performance difference with [10] under 802.16 m environments. The results show that our methodology enhances overall system throughput by eliminating radio resource waste.

## Figures and Tables

**Table 1:** MCS and number of users supporting them.

	*m*			
	1	2	3	4
Modulation	BPSK	QAM16	QAM64	QAM256
Code rate	3/4	1/2	2/3	3/4
Net PHY bit rate (Mbps)	2.12	5.64	11.29	13.39
The number of users supporting MCS	14	13	8	7

**Table 2:** Slot size of used solution and the comparison of packet number between them.

	*m*			
	1	2	3	4
Slot size of the proposed solution (*μ*s)	28.29	10.62	5.28	4.47
Slot size of fixed-slot solution (*μ*s)	28.29	28.29	28.29	28.29
The number of packets which the proposed method can transmit during a slot of the fixed-slot method	1	2.67	5.33	6.33

**Table 3:** Performance difference between them.

	*m*			
	1	2	3	4
The sum of utilities of the proposed solution	7478			
Total used time in a frame	0.0299994			
The proposed solution (ms)	11.316	8.496	8.131	2.056
The number of packets of the proposed solution	400	800	1540	460

The sum of utilities of fixed-slot solution	6950			
Total used time in a frame	0.02979864			
Fixed-slot solution (ms)	11.31	5.658	9.051	3.771
The number of packets of fixed-slot solution	400	400	1600	800
